# Body Height of Elite Basketball Players: Do Taller Basketball Teams Rank Better at the FIBA World Cup?

**DOI:** 10.3390/ijerph17093141

**Published:** 2020-04-30

**Authors:** Ivan Zarić, Filip Kukić, Nemanja Jovićević, Milan Zarić, Milan Marković, Lazar Toskić, Milivoj Dopsaj

**Affiliations:** 1 Faculty of Sport and Physical Education, University of Belgrade, 11000 Belgrade, Serbia; jovichevich@gmail.com (N.J.); info@licnitrener.com (M.Z.); mm_milanm@yahoo.com (M.M.); milivoj.dopsaj@fsfv.bg.ac.rs (M.D.); 2 Police Sports Education Center, Abu Dhabi 253, UAE; filip.kukic@gmail.com; 3 Faculty of Sport and Physical Education, University of Priština, 38220 Kosovska Mitrovica, Serbia; lazartoskic@gmail.com; 4 Institute for Sport, Tourism and Service, South Ural State University, 454080 Chelyabinsk, Russia

**Keywords:** performance modelling, selection, talent acquisition, anthropology, morphology

## Abstract

Body height is among the most important attributes of basketball players. Whether it differs among the basketball players who compete at the world basketball championship (FIBA-WC) is unknown. The aim of this study was to investigate the differences between the basketball players from the teams ranked 1–16 and those ranked below 16th place. The body heights of all players from the last three FIBA-WCs were collected and allocated according to the ranking at the FIBA-WC and analyzed by position in team. An independent sample t-test was conducted to analyze the difference in body height of players ranked 1–16 and players who ranked below 16th place. The players from the first 16 teams were significantly taller at three positions: point guards (Difference = 2.92 cm, *p* < 0.05), shooting guards (Difference = 2.16 cm, *p* < 0.05), and small forwards (Difference = 2.49 cm, *p* < 0.01). Body height seems to be an important factor for the performance of the basketball team at the FIBA-WC. Considering that all players at the FIBA-WC went through rigorous selection process to be in their national teams, body height of the higher-ranked players could be used as a reference value.

## 1. Introduction

Basketball has been among the most popular sports in the world [1] whereby the finals in basketball are the closing event of the Olympic games. It is a dynamic game where two teams of five players each run back and forth attacking and defending a basket set at the height of 3.05 m. The game is characterized by high-intensity intermittent runs, often requiring planned or unpredictable changes of direction, a variety of specific technical skills and well-developed jumping ability [2,3,4]. Thus, a player’s achievement and success depends on physical abilities, physiological profile, mental abilities and tactical skills as well as on their anthropometric characteristics such as body height and body weight [5,6,7,8]. However, considering the height of the basket and that the winner is who scores more baskets, body height and weight have had the highest priority during the selection process and when establishing an in-court position [9,10].

When investigating the differences in high level basketball players, Pehar et al. [7] found that players from the first division were significantly taller than the players from the second division. Furthermore, Garcia-Gil et al. [11] conducted regression analysis and found that body height was among the main predictors of performance index rating among elite female basketball players. Moreover, authors reported that the performance index rating correlated with the arm span and contracted arm perimeter. This could be also attributed to body height as taller people normally have longer hands, which reflects in longer arm span [12]. These characteristics are of importance in basketball game during jumping (i.e., rebounding and blocking), defending the space (i.e., covers wider and higher space), and shooting or dunking (i.e., over shorter players). Thus, body height provides the advantage in every aspect of the game [13].

Although the evidence suggests that teams with taller players may have better results, the existing studies mostly covered individual national basketball leagues. For the world cup (FIBA-WC), national teams select only the 12 best players that the country has. Moreover, the countries need to qualify for the world championship as strictly defined number of countries can participate. Accordingly, it could be assumed that those players who come to the FIBA-WC are the most elite in their countries and in the world. The competition system at FIBA-WC was set by International Basketball Federation (Fédération Internationale de Basketball [FIBA]). It consists of group phase and knockout phase. In a group phase at 2010 and 2014 FIBA-WC, six teams within one group played between each other and the best two teams from each group went into the knockout phase. At 2019 FIBA-WC, four teams in eight groups played against each other in the first group phase and two teams from each group went to second group phase. The second group phase consisted of four groups with four teams each and again two teams from each group went to knockout phase. The knockout phase means that every game until the end of the championship is played by the rule who win goes further into competition, until the last final game where two best teams play for the first place. Considering the nature of the knockout phase and the quality of teams that entered, where the winner can be any team, it could be assumed that the teams from the knockout phase are in some characteristics better than the teams that did not pass to this phase.

Considering this, the question arises whether the players from better ranked teams (i.e., knockout phase) are significantly taller than those whose teams ranked lower (i.e., qualified but did not pass to knockout phase). Therefore, this study aimed to evaluate if the teams from the knockout phase of the world basketball championship were significantly taller that the teams who did not enter the knockout phase. It is hypothesized that the teams and players who went through the first phase of FIBA-WC are significantly taller than those drop-off the competition in earlier stages. This would provide a position-specific body height model of the players from the teams that ranked higher at the FIBA-WC.

## 2. Materials and Methods

### 2.1. Participants

A retrospective analysis of body height for 960 elite basketball players from national basketball teams that competed at the last three (2010, 2014, and 2019) FIBA-WC. Body heights were obtained from the official web page of International Basketball Federation (Fédération Internationale de Basketball [FIBA]). Twenty-four teams competed at the 2010 and 2014 FIBA-WC, while the rules were changed for the 2019 where 32 teams were allowed to compete. The study is conducted in accordance with the Helsinki declaration regarding the ethical principles for medical research involving human subjects [14]. The study design was approved by the Ethical Board (number 484-2) of the Faculty of Sport and Physical Education, University of Belgrade, Belgrade, Serbia.

### 2.2. Procedures

National teams provided body heights of players to FIBA with registration of the team. How each team obtained body heights of their players could not be defined for this research. However, considering the importance of competition such as the FIBA-WC, it is reasonable to assume that the professionals who did the measurements provided accurate and reliable data. The teams from three FIBA-WCs were divided into two groups according to their ranking. The first group included the teams that passed the group phase at 2010 and 2014 FIBA-WC and the first group phase at 2019 FIBA-WC. These were the teams ranked from first to 16th place and this group was named 1–16 and had mean age 29.3 ± 2.7 years. At the 2010 and 2014 FIBA-WC, after the first group phase teams entered into the knockout phase, while in 2019 after the first group phase 16 teams entered into second group phase and eight teams entered the knockout phase. This difference occurred due to the change of rules as there were eight teams more at 2019 than at the 2010 and 2014 FIBA-WCs. However, considering that all teams had to go through the prequalification for the FIBA-WC and then through the first group phase of the FIBA-WC, it could be considered that the 16 teams that passed these stages were a homogenous group, thereby had players with similar skills and body characteristics. The second group (16 >) consisted of teams that did not pass the group phase of the competition and therefore were ranked below the 16th place. The mean age of this group was 28.9 ± 2.8 years. Although all teams that qualified to the FIBA-WC consist of rigorously selected and professionally trained athletes, it could be assumed that those who do not pass the first group phase are to some degree different in some characteristics compared to those who do pass it. For each group, body height was analysed relative to a player’s position in the team and compared between the groups. The positions of players (point guard, shooting guard, small forward, power forward and center) were also collected from the FIBA web page, where each player has a profile with information about the position. For guards and forwards, whose position was not defined precisely (i.e., point or shooting guard and small or power forward) at the web page, authors conducted the video analysis of two to three different game plays [15] following procedures explained elsewhere [16]. The rationale was to determine if the point guards, shooting guards, small forwards, power forwards and centers from the 1–16 group were taller than those below the top 16 group.

### 2.3. Statistical Analyses

The statistical analyses were conducted using the Microsoft Excel and Statistical Package for Social Sciences (SPSS, version 22.0). The data were analysed descriptively for mean, standard deviation (SD), minimum (MIN), maximum (MAX), coefficient of variation (CV%), and 95% confidence interval (95% CI). The Kolmogorov–Smirnov test was used to evaluate the normality of distribution. The differences between the groups were analysed by an Independent sample t-test, with the significance level set at *p* < 0.05. The parametric test was chosen because the sample size was large enough and non-parametric tests in large studies may provide answers to the wrong question [17,18]. The Cohen’s effect sizes were calculated for the magnitude of differences between the groups [19] using the formula: (M_2_−M_1_)/SD, where M_1_ and M_2_ were the means of the groups, while SD was a pooled standard deviation of compared groups. The magnitudes of differences were defined as small = 0.2–0.5, moderate = 0.5–0.8, and large ≥ 0.8. The body height model of the most elite players was analysed descriptively and the range was presented in quartiles.

## 3. Results

The descriptive statistics for each position at the three FIBA-WC are shown in Table 1. Considering the whole sample, the mean body height for each position across all three competitions. Kolmogorov-Smirnov test that data at each position was normally distributed, with minimal *p* = 0.200.

**Figure 1:**
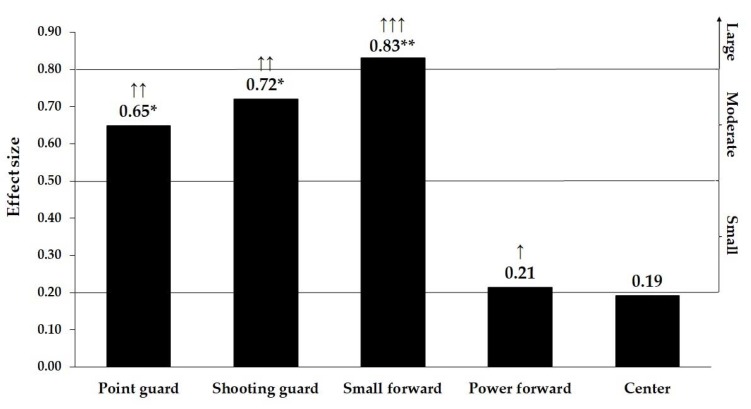
The size of the difference in body height between the teams of different ranking relative to position in team. * Significant at *p* < 0.05, ** Significant at *p* < 0.01, ↑—small effect size, ↑↑—moderate effect size, ↑↑↑—large effect size.

However, once split into groups relative to the team ranking, the body height of point guards, shooting guards, and small forwards from the teams ranked 1–16 were significantly higher than the height of those ranked below 16th place (Table 2). The body height of power forwards and centers were similar, regardless of the team ranking. Moreover, the analysis of the effect sizes revealed a moderate to large magnitude of difference between the groups (Figure 1), whereby the biggest difference occurred at the position of small forward. Although the observed difference in height was not statistically significant in power forwards, the effect size analysis revealed the difference of small magnitude (Figure 1).

Finally, considering that the heights of the 1–16 group were significantly higher, the descriptive statistics and quartile analysis are presented in Table 3.

## 4. Discussion

The aim of this study was to investigate if players from the teams ranked 1–16 were taller than players from the teams ranked below the 16th place, whereby differences in body height were investigated at each position. The main findings of this study indicate that teams with taller players on certain positions were ranked higher at the FIBA-WC. Namely, point guards, shooting guards, and small forwards of the first 16 teams were significantly taller than the players at the same position from the teams ranked below the 16th place. The obtained differences were small to large in magnitude, indicating the importance of body height in the selection process for the elite level of play.

The descriptive statistics consistently showed that starting from the position of point guard towards center, body height gradually increases. Each position has a specific set of tasks that in a large measure determine the body height of the players. For example, point guards are playmakers who dribble the ball the most. They are chased by defenders in order to disrupt the planned action, which is why they need to be quick and agile with remarkable ball control. High proficiency in agility while dribbling the ball under pressure allows them to focus to what is happening on the court so they can employ their teammate. In that regard, players with lower center of body mass are well suited to quickly accelerate, decelerate and possess good agility. Thus, point guards are usually somewhat shorter (although taller comparing to general adult male population [20,21,22]) than the other positions. Positions of shooting guards and small forwards have a lot of running tasks without the ball as they are opening or closing the space for the action to be completed, whereby shooting guards may move to the position of point guard if needed. Thus, although the shooting guards and small forwards are both taller than the point guards are, the shooting guards are shorter than the small forwards. Finally, power forwards and centers are tall and strong, ready to block the player or shoot, or jump for the ball under the basket (centers) or little further from the basket (power forwards). Additionally, when close to the basket they often play in close contact, pushing the opponent towards (offence) or off (defense) the basket, where the height and body weight have been advantage.

The results of the t-test are in accordance with previous studies that found body height be a significant predictor of basketball performance [4,5,7]. However, given that the success in basketball performance includes multiple factors whose magnitudes of influence may vary between players, it is hard to precisely define the magnitude to which the body height contributes to it. The importance of the height reflects in the fact that the players at the positions of point guards, shooting guards, and small forwards from the teams ranked first to 16th place were significantly higher than the players on the same positions who ranked below the 16th place. In addition, there was no significant difference in the body height of centers and power forwards, regardless of the team ranking, indicating that all national teams that qualified to the FIBA-WC selected the tallest centers from their countries. Typically, these are people who are in the highest percentiles or extremes of the population and they do not differ among themselves, regardless of the origin [20,21]. The practical advantage of tall players reflects in high blocking reach, wide space covered by arm span, high body weight and absolute strength, which all contribute to successful one-on-one play under the 3.05-m basket [23]. In a basketball game, the main jobs of center and power forwards are screens, blocking, collecting the balls (rebounds), and scoring under the basket, whereby power forwards often shoot very effectively from the distance as well. Indeed, some centers and power forwards (very few) move extraordinarily and do more than previously stated, but this only additionally strengthens the importance of the related body height and dimensions as these are usually hardly stoppable players who fill all the columns of the statistics (i.e., often score triple-double). In that regard, the results suggest that even among point guards, shooting guards, and small forwards of a similar skill level, those who are taller are a better fit for the more successful teams (i.e., perform better).

It could be argued that they are physically more dominant in one-on-one play offensively and defensively, that they have higher ball releasing point while shooting or in defensive jumping, and their arm span may cover wider space when guarding the space. When a player is covered by an aggressive defender, his aim is to perform the shot at the highest possible release point. The two-legged jump shot may amount to over 70% of all the shots during a game, whereby greater performance in executing the jump shot depends on the height at which the ball is released [13]. Thus, a taller point guard, shooting guard, and small forward may have advantage both ways, higher releasing point or higher reach while blocking the shot. Note that the first four players in the history of NBA by number of triple-doubles are point guards whose heights were 191–206 cm. Considering the tactical standpoint, after the screen, point guards, shooting guards or small forwards often need to defend power forwards and centers. Coaches may try to exploit the “weakness” of the opposing team with shorter guards or small forwards by frequently using pick-and-roll when centers or power forwards would play one-on-one against significantly shorter players. Similarly, when power forwards are good at shooting from distance, they tend to play pick-and-pop, which may be more comfortable if they are taller than the player in defense. In contrast, taller players in defense may have higher chances to be more successful when defending the opponents. For example, it would not be the same if the point guard of 180 cm defends the power forward of 204 cm after pick-and-pop as it would be if point guard is 195 cm. In addition, a small forward of 205 cm would have advantage in low post play against the small forward of 198 cm, which a coach could tactically exploit by setting the actions so the taller small forward has the space to play offensively more often by either shooting over the defendant or pushing him under the basket. In addition, taller players with longer extremities exit the off-ball screen very far, which provides them with longer time to shot, which may contribute to shooting accuracy.

From the quartile analysis of body height, overlapping could be observed between the positions. It seems that some point guards and shooting guards may have the same body height, thus play either position if they are also skilled for it; these positions may belong to the first body height cluster. The body height of taller small forwards overlaps with shorter power forwards, indicating that skilled players on these positions of similar body height may be used in the game on both positions, depending on the team tactics. This could be the second body height cluster. Similarly, body height of taller power forwards overlaps with shorter centers, which indicates that players on these two positions who have similar body height and adequate skill level can replace each other in the game if needed, forming the third body height cluster. Considering this, it could be concluded that at each position could be divided into three subclassifications, short, average, and tall. Short point guards (<185 cm), average point guards (185–191 cm), tall point guards (≥192 cm); short shooting guards (<191 cm), average shooting guards (191–195 cm), and tall shooting guards (≥196 cm); short small forwards (<200 cm), average small forwards (200–203 cm), and tall small forwards (≥204 cm); short power forwards (<204 cm), average power forwards (204–207 cm) and tall power forwards (≥208 cm); short centers (<208 cm), average centers (208–211 cm), and tall centers (≥212 cm).

This study is not without limitations. Body height certainly is not the only factor important in talent acquisition and the selection process, nor could this study define whether body height is the most important factor. In that regard, the inclusion of variables such as players’ age, body mass, body composition (i.e., percent skeletal muscle mass and body fat), physical abilities and specific basketball performance should be included for a more comprehensive analysis. However, the study clearly shows that body height is an important factor for teams ranking at FIBA-WC, thus providing the guidance to talent acquisition specialists and coaches.

Considering this, body height could be considered an important factor of success in basketball and, given that the players from the first 16 teams were significantly taller, their body height could be used as the reference values for the selection. This is further reinforced by the fact that the average height of the players from the National Basketball Association (NBA) is the same (200.6 cm) as of the average height of the payers from the first 16 teams [24]. Furthermore, the average height of the Spain national basketball team for the 2008 Olympics was 199.2 cm, while the average body height of the best Serbian national league in 2007 was 199.5 ± 7.4 cm [24]. It is of note that the best players from each of the mentioned leagues are normally the representatives of their countries on the FIBA-WC. Moreover, the players at the FIBA-WC are already rigorously selected by professional peers such as coaches and scouts. Therefore, the results obtained in this study seem to accurately describe the body height of the most elite basketball players in the world, thereby defines the reference norms for the player selection. This may have practical implications in modeling a successful basketball team and player positioning.

## 5. Conclusions

The body height of basketball players seems to play the major role in their overall performance at all positions. It could be concluded that, among the point guards, shooting guards, and small forwards of the same basketball knowledge (i.e., tactics) and skill, those who are taller perform better. On the other hand, when it comes to power forwards and especially centers who are typically chosen for their height, those who possess better basketball knowledge and skill perform better. Therefore, considering that body height is genetically predetermined and cannot be altered by training, it needs to be the part of selection process and talent acquisitioning for the elite level of basketball. This does not necessarily mean automatic exclusion of shorter players based on height. If they perform as same as or better than the taller player at the same position and contribute to the overall team performance to a larger extent, body height should not be the limiting factor. However, reference values for body height set based on the data from the elite basketball players of the world provide a valid body height model of high performers at each position within the team. In addition, it provides an insight into body height clusters that address possible overlapping of players at different positions. Although in practice coaches sometimes use the same player in different positions (i.e., shooting guard replacing point guard), body height has not been modeled so far in such a way to cover this space. Therefore, this study provides a formal and practical position-specific body height model of elite basketball players.

## Figures and Tables

**Table 1:** Body height of basketball players from three FIBA-WCs relative to position in team.

**World Basketball Cup 2010**	**Positions**	**N**	**Mean (cm)**	**SD**	**CV%**	**Min**	**Max**	**95% CI**	
								**Lower**	**Upper**
	1-Point guard	30	189	5	3	178	199	187	191
	2-Shooting guard	91	192	6	3	176	206	191	193
	3-Small forward	82	202	4	2	192	213	201	203
	4-Power forward	20	205	3	2	198	211	204	207
	5-Center	65	210	5	2	198	222	209	211
**World Basketball Cup 2014**	**Positions**	**N**	**Mean (cm)**	**SD**	**CV%**	**Min**	**Max**	**95% CI**	
								**Lower**	**Upper**
	1-Point guard	47	188	6	3	170	198	186	189
	2-Shooting guard	70	192	6	3	180	204	191	194
	3-Small forward	67	201	5	3	189	213	200	202
	4-Power forward	48	205	4	2	198	215	204	207
	5-Center	56	210	4	2	200	218	209	211
**World Basketball Cup 2019**	**Positions**	**N**	**Mean (cm)**	**SD**	**CV%**	**Min**	**Max**	**95% CI**	
								**Lower**	**Upper**
	1-Point guard	67	188	6	3	176	201	187	190
	2-Shooting guard	90	192	5	3	179	209	191	193
	3-Small forward	91	201	5	2	189	211	200	202
	4-Power forward	59	205	3	2	195	210	204	205
	5-Center	77	209	5	2	197	222	208	210

**Table 2:** Differences in body height of players from the first 16 teams and below the 16^th^ place, relative to position in team.

Positions	Ranking (1–16)	Ranking (below 16)	Mean Difference
	Mean ± SD	Mean ± SD	
1-Point guard	189 ± 5 cm	186 ± 4 cm	2.92 *
2-Shooting guard	193 ± 3 cm	191 ± 3 cm	2.16 *
3-Small forward	202 ± 3 cm	200 ± 3 cm	2.49 **
4-Power forward	206 ± 3 cm	205 ± 3 cm	0.64
5-Center	210 ± 4 cm	209 ± 3 cm	0.67

* Significant at *p* < 0.05, ** Significant at *p* < 0.01.

**Table 3:** Descriptive statistics for each position for the teams that ranked 1–16.

Positions	Mean (cm)	SD	CV%	Min	Max	Quartiles		
						25th	50th	75th
1-Point guard	189	5	3	178	199	185	190	192
2-Shooting guard	193	3	2	185	200	191	194	196
3-Small forward	202	3	2	194	210	200	202	204
4-Power forward	206	3	1	200	212	204	206	208
5-Center	210	4	2	200	216	208	210	212
All positions	200	9	4	178	216	198	200	202
